# Hypoglycemic and Hepatoprotective Activity of Fermented Fruit Juice of *Morinda citrifolia* (Noni) in Diabetic Rats

**DOI:** 10.1155/2011/875293

**Published:** 2010-10-14

**Authors:** B. Shivananda Nayak, Julien R. Marshall, Godwin Isitor, Andrew Adogwa

**Affiliations:** Department of Preclinical Sciences, Faculty of Medical Sciences, The University of the West Indies, St. Augustine, Trinidad and Tobago

## Abstract

*Morinda citrifolia* is a medicinal plant used to treat diabetes and liver diseases. The fermented fruit juice of the *M. Citrifolia* (optical density = 1.25) was used to study the hypoglycemic and hepatoprotective properties in diabetes-induced rats. The rats were randomly distributed into 4 groups (control, diabetic experimental, diabetic standard, and diabetic untreated) of 6 each. Diabetes was induced by administering Streptozotocin (50 mg/kg body weight). Fasting blood glucose, body mass, liver tissue glycogen content, and the extent of liver degeneration were assessed. Diabetic experimental animals were treated with *M. citrifolia* juice (2 ml/kg, twice a day) and diabetic standard with reference hypoglycemic drug, glibenclamide orally for 20 days. Both the groups exhibited a significant reduction in blood glucose level of 150 mg/dl ±15.88 and 125 mg/dl ±3.89, respectively, as compared to diabetic untreated with FBS = 360.0 mg/dl ±15.81, (*P* < .003). On 10th day of experiment, diabetic experimental animals exhibited a decrease in body mass (10.2 g, 5.11%) which increased significantly by the 20th day (6 g, 3.0%, *P* < .022). Histological study of liver tissue obtained from untreated diabetic animals revealed significant fatty degeneration as compared to other three groups. The data of this study proved the hypoglycemic and hepatoprotective activity of *M. citrifolia*.

## 1. Introduction

Diabetes mellitus (DM) is a group of metabolic disorders characterized by hyperglycemia, with disturbances of carbohydrate, fat, and protein metabolism resulting from defects in insulin secretion, insulin action, or both. Regulation of the major metabolic pathways involved in fat, carbohydrate, and protein is of critical importance to bodily function and is achieved by several hormones. Insulin, a pancreatic hormone, is essential in the regulation of carbohydrate, lipid, and protein metabolism. It functions to preserve and to create energy reserves in the body by inhibiting catabolic processes such as lipolysis, gluconeogenesis, proteolysis, and glycogenolysis. Insulin promotes glycogenesis, fatty-acid synthesis, intestinal amino-acid uptake, and plasma glucose uptake [1, 2].

Underproduction of, or insensitivity of cells to insulin or a combination of, both represents the core aetiology of diabetes mellitus. Management of diabetes mellitus is based upon mechanisms which increase insulin secretion. Secretagogues sensitize cells to insulin, and sensitizers inhibit intestinal glucose absorption and reduce gastric emptying. Current treatment methods, although quite effective, can have undesirable side effects. Additionally, continuous use of the medications listed above may constitute an economic burden on the user [3].

Natural remedies from medicinal plants are considered to be effective and safe alternative treatment for hyperglycemia and liver toxicity. There is a growing interest in herbal remedies because of their effectiveness, minimal side effects in clinical experience, and relatively low cost. Herbal drugs or their extracts are prescribed widely, even when their biological active compounds are unknown [4]. Therefore, studies with plant extracts are useful to know their efficacy and mechanism of action and safety. 

In Trinidad and Tobago, a large variety of plants have been employed by contemporary traditional healers in the management of diabetes based on their received medicinal benefit. These include *Momordica charantia *(Caraaili),* Carica papaya *(Paw-paw)*, Catharanthus roseus *(white periwinkle), *Neurolaena lobata* (Zebapique), Cecropia peltata (trumpet Bush), *Cordia curassavica* (Black Sage), and *Bontia daphnoides *(Olive Bush). Users of these plants have claimed significant therapeutic benefits that, at times, may even exceed the clinical benefits of modern drugs [5, 6].

*Morinda citrifolia* L (Rubiaceae) also known as noni, or Indian mulberry, is a small evergreen tree. It is native to the Pacific islands, Polynesia, Asia, and Australia. The leaves are 8 or more inches long and are oval in shape. The fruit is 3 to 4 inches in diameter with a warty, pitted surface. *M. citrifolia* is one of the most important traditional Polynesian medicinal plants and has been heavily promoted for a wide range of uses; from arthritis and burns to circulatory weakness, diabetes, cancer, skin inflammation, and wounds [7–10]. 

## 2. Methods

### 2.1. Preparation of Aqueous Fermented Extract

Freshly harvested ripe noni fruits were collected from a local supplier. The fruit was thoroughly washed in luke warm water to remove fungus and to retard the growth of microbiological organisms that may be sensitive to heat. The noni fruit was then cut into pieces to fit into a 2000 ml container and 500 ml of water was then added. The chunked (noni) fruit was left to ferment for 6–10 weeks at room temperature. At the end of the fermentation period, 400 ml of stock (noni) ferment was transferred to a 1000 ml container. The 400 ml stock ferment was filtered using Whatman filter paper and vacuum filtration to eliminate debris and fruit particles from the stock solution. Thirty millilitres of the filtrated ferment was used for qualitative phytochemical analysis and 10 ml was used for UV/VIS spectroanalysis and optical density determination. The remaining 360 ml was used for the experiment.

### 2.2. Experimental Design

Healthy Sprague-Dawley male rats weighing between 200 and 220 g were used for the study. The animals were individually housed and maintained on normal food and water *ad libitum*. Animals were periodically weighed before and after experiments. Fasting blood glucose levels of the animals were assessed using the glucometer, which employed the glucose oxidase/peroxidase reaction. Blood for glucose estimation was obtained from the tail veins of the rats. All animals were closely observed for any infection, and those that showed signs of infection were separated and excluded from the study. The study was approved by the Ethics Committee of the (EC-A/5-2009) Faculty of Medical Sciences, The University of the West Indies, St. Augustine. The rats were randomly distributed into 4 groups of 6 each as follows:

- normal control group: this group received only food and water,
- diabetic experimental group: In addition to food and water, this group received fermented juice of *M. citrifolia* (OD = 1.2470 at 338 nm) orally at a dose of 2 ml/kg twice daily for 20 days from the day of diabetes induction,
- diabetic standard group: in addition to food and water, this group received glibenclamide, dissolved in DMSO at a dose of 2.0 mg/kg/day orally for 20 days,
- diabetic untreated group**: **this group of rats was provided food and water.

All the groups were provided food and water *ad libitum.*

### 2.3. Induction of Diabetes Mellitus

Diabetes was induced by administering 50 mg/kg of Streptozotocin in cold citrate buffer, pH 4.5, intraperitoneally to overnight fasted adult Sprague-Dawley rats. After three days, animals with a fasting blood glucose >200 mg were considered to be diabetic.

### 2.4. Histological Study

Liver was obtained on day 20 from all the groups of animals for the histological study. The tissues were fixed in 10% buffered formal saline and processed for routine histological evaluation. Sections of 7.0 micron were cut from the tissues, some of which were stained with Periodic Acid Schiff (PAS) and the rest with Haematoxylin and Eosin.

### 2.5. Statistical Analysis

The means of fasting blood glucose and body mass between groups at different time intervals was compared using One-way ANOVA, descriptive test, followed by Dunnett's post hoc test. Data was analyzed using the SPSS (Version 16.0, Chicago, USA). Differences between groups were considered significant at *P* <  .05 levels.

## 3. Results

### 3.1. Phytochemical Analysis

Qualitative phytochemical analysis of fermented juice of *M. citrifolia* revealed the presence of saponins, triterpenes, steroids, flavonoids, and cardiac glycosides.

### 3.2. Fasting Blood Glucose

On the 20th day, after treatment, there was a significant normalization of fasting blood sugar, observed in diabetic experimental animals treated with *M. citrifolia* and the diabetic standard with reference hypoglycaemic drug, glibenclamide as compared to diabetic untreated animals. In diabetic experimental animals, there was a significant decrease in fasting glucose from an excess of 300 mg/dl (day 3) to 150 mg/dl (day 20), and this represented a decrease of 52.6%. The diabetic standard group showed the reduction in fasting blood sugar from 250 mg/dl to 125 mg/dl. The decrease in fasting blood glucose levels was not observed with the untreated diabetic animals (Figure 2).

### 3.3. Body Mass Variation

**Figure 1:**
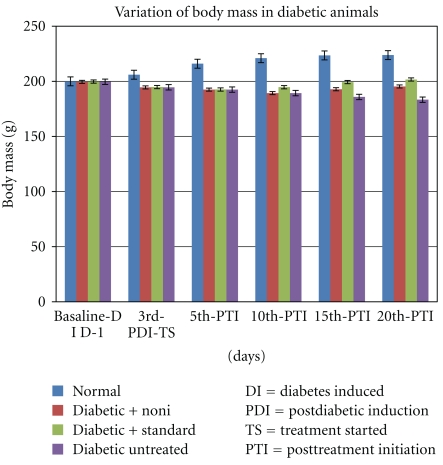
Body mass of normal control, diabetic experimental, diabetic standard, and diabetic untreated measured at basal level (before treatment) and during 20 days of drug treatment; (*n* = 6) each column represents mean ±SE. (*P < .022) versus diabetic untreated (One-way ANOVA, descriptive test, followed by Dunnett's multiple comparison post hoc test).

**Figure 2:**
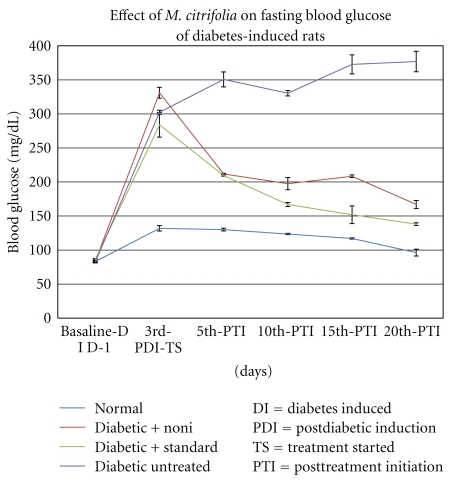
Fasting blood glucose of normal control, diabetic experimental, diabetic standard, and diabetic untreated measured at basal level (before treatment) and during 20 days of drug treatment; (*n* = 6) each line represents mean ±SE. (*P < .003) versus diabetic untreated (One-way ANOVA, descriptive test, followed by Dunnett's multiple comparison post hoc test).

In the latter 10 days of the treatment period, diabetic experimental and diabetic standard group animals exhibited a significant increase in body mass of 3.0 and 8.0 percent, respectively, whereas the diabetic untreated animals showed a decrease in body mass, (16.3 g, 8.17%) over the course of the 20-day treatment period (Figure 1) (*P* < .022).

### 3.4. Histological Analysis

**Figure 3:**
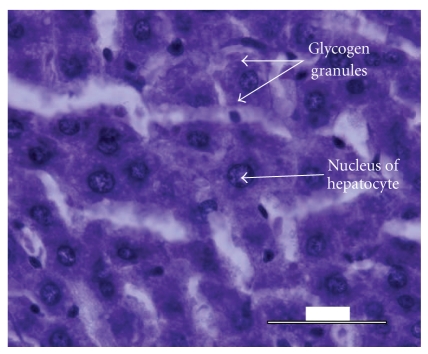
Histology of the liver specimen obtained from normal control animal (PAS-stain). No fatty infiltrates were observed in the liver specimen above. Glycogen granules were well distributed thoroughout the slide area.

**Figure 4:**
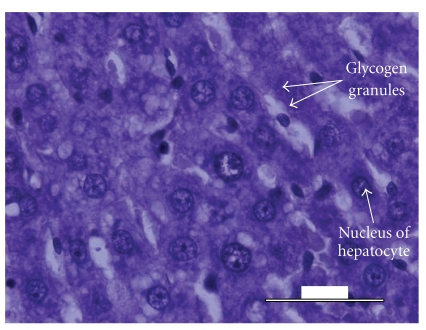
Histology of the liver specimen obtained from *M. Citrifolia*-treated animals (PAS-stain). Few small fatty Infiltrates were observed in the liver specimen above. Glycogen granules were well distributed thorough-out the slide area.

**Figure 5:**
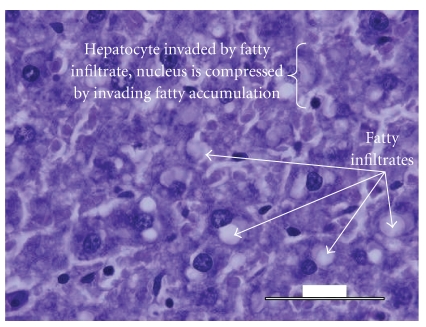
Histology of the liver specimen obtained from untreated diabetic animals (PAS-stain). Numerous large fat cells were seen in the cytoplasm of numerous hepatocytes. Glycogen deposition was sparse and irregular. Morphology of hepatocyte was also altered by the presence of the fatty globules.

**Figure 6:**
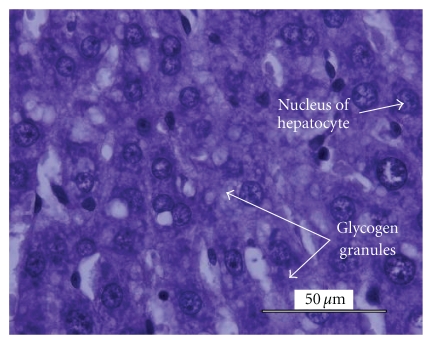
Histology of the liver specimen obtained from diabetic standard animals (PAS-stain). Well-organised glycogen granules were seen.

Histological analysis of the liver tissue obtained from the untreated animals revealed a significant fatty degeneration (Figure 5), as compared to the liver tissue obtained from the normal control (Figure 3) and diabetic experimental animals treated with* M. citrifolia* (Figure 4). Hepatocytes of the untreated diabetic animals were irregularly shaped, and numerous large fatty infiltrates were seen (Figure 5) in the cytoplasm of 35% of those liver cells as compared to the normal control (Figure 3), diabetic experimental (Figure 4), and diabetic standard animals (Figure 6).

## 4. Discussion

Fasting blood sugar in diabetic rats represents an important indicator of diabetic status. The results of this study clearly indicate that the effect of *M. citrifolia* fruit juice is equivalent to the reference oral hypoglycemic drug, glibenclamide. Indeed, both treatments significantly reduced hyperglycemia. The constituents of noni juice might have lowered glucose levels either by promoting insulin secretion like sulphonylurias or gliptines or by increasing insulin receptor sensitivity like biguanides and glitazones. Recently, scientists showed a similar phenomenon of hypoglycaemic action with the extracts of *Argania spinosa* and *C. Dactylon* [11, 12].

 Animals treated with fermented noni fruit juice exhibited (3.0%) increase in body mass in the final 10 days of treatment. Conversely, untreated diabetic animals demonstrated a (8.17%) decrease in body mass over the same period of time. Untreated diabetic animals were consuming more amount of water when compared to the noni juice-treated rats. 

Diabetic rats treated with noni juice had reduced the hepatocyte fatty degeneration (fatty globules were smaller and less numerous) when compared to diabetic untreated animals. This suggests a possible hepatoprotective property of the fermented noni juice. Wang et al. demonstrated the hepatoprotective activity of noni fruit juice against CCL4-induced liver damage [13]. Recently, researchers showed that the Indian honey protects liver against oxidative damage and it could be used as an effective hepatoprotector against Acetaminophen-induced liver damage [14]. The liver protecting activity was well correlated with its antioxidant properties. After analyzing our results and previous reports on *M. citrifolia*, we hypothesize that the hepatoprotective activity of *M. citrifolia* is due to its possible antioxidant properties of its flavonoid constituents. 

**Figure 7:**
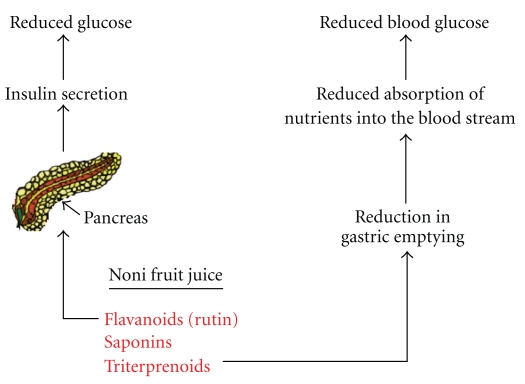
Schematic diagram showing the possible role of noni fruit juice constituents in reducing blood sugar.

The hypoglycaemic activity, exhibited by the noni fruit ferment, may be attributed to the presence of triterpenes and saponins. Researchers demonstrated the presence of a significant quantity of bioactive compounds like flavonoids, triterpenoids, triterpenes, and saponins in *Morinda citrifolia* [15, 16]. It has been suggested that saponins may significantly inhibit gastric emptying [17, 18]. The saponins could inhibit gastric emptying either by promoting secretion of glucagon like peptides-1 (GLP-1) or by inhibiting its degradation. This drug-induced gastroparesis is an effective method of managing hyperglycemia because it slows the process of nutrient absorption into the blood stream. Also, the presence of saponin in *M. citrifolia* may have a glucagon decreasing effect and may enhance glucose utilization lowering blood glucose. It is also reported that saponin stimulates insulin release from the pancreas [19] (Figure 7), and it could be due to decreased degradation of glucagon like peptides. On the other hand, glibenclamide exerts hypoglycemic action by stimulation of insulin secretion and inhibition of glucagon release. The remaining intact pancreatic cells are stimulated by *M. citrifolia* or glibenclamide, and the serum insulin level is increased, and the blood glucose is decreased. Rutin is a flavonoid (a glycoside composed of rutinose and quercetin) found in significant quantities in the noni fruit, and it is postulated that the rutinose residues may act as a secretagogue, which potentiates insulin secretion by a mechanism related to that of sugar sucrose. Triterpenoids have also been indicated as possible therapeutic agents that can be beneficial in the management of diabetes mellitus, as they have been shown to be effective in improving symptoms of glycosuria and blood sugar in alloxan-induced mice [20, 21]. The above are preliminary indications, and further detailed studies are necessary to find out whether the action of the fruit juice is due to one or more of the above-mentioned possible mechanisms or not. Thus, the fermented fruit juice of *M. citrifolia *seems to be useful in controlling blood sugar and hepatic injury. Purification of noni fruit juice and identification of the active principle may yield a good hypoglycaemic and hepatoprotective drug.

## 5. Conclusions

Data of this study showed that fermented juice of *M. citrofolia *may possess hypoglycemic and hepatoprotective properties based on the parameters examined. *Morinda citrofolia *significantly improved the fasting glucose status of diabetic animals over the period of twenty days. There was also an observed increase in body mass. It can be presumed that *M. citrofolia *either potentiates the action of insulin directly or that it increases peripheral tissue sensitivity to the storage hormone. It may have the capacity to improve fat metabolism and as such reduce fatty accumulation in the liver. Nonetheless, clinical studies are required to assess the potential benefit of *M. citrofolia *preparations in humans.

## References

[1] World Health Organization (1999). *Department of Non-Communicable Disease Surveillance. Definition, Diagnosis and Classification of Diabetes Mellitus and its Complications*.

[2] Nelson LD, Cox MM (2004). *Lehninger’s Principles of Biochemistry*.

[3] Grahame-Smith DG, Aronson JK (2002). *Clinical Pharmacology and Drug Therapy*.

[4] Gupta RK, Kesari AN, Murthy PS, Chandra R, Tandon V, Watal G (2005). Hypoglycemic and antidiabetic effect of ethanolic extract of leaves of *Annona squamosa* L. in experimental animals. *Journal of Ethnopharmacology*.

[5] Lans CA (2006). Ethnomedicines used in Trinidad and Tobago for urinary problems and diabetes mellitus. *Journal of Ethnobiology and Ethnomedicine*.

[6] Mahabir D, Gulliford MC (1997). Use of medicinal plants for diabetes in Trinidad and Tobago. *Pan American Journal of Public Health*.

[7] Elkins R (1997). *Hawaiian Noni (Morinda citrofolia)*.

[8] Hirazumi A, Furusawa E, Chou SC, Hokama Y (1996). Immunomodulation contributes to the anticancer activity of Morinda citrifolia (Noni) fruit juice. *Proceedings of the Western Pharmacology Society*.

[9] Nayak BS, Sandiford S, Maxwell A (2009). Evaluation of the wound-healing activity of ethanolic extract of *Morinda citrifolia* L. leaf. *Evidence-Based Complementary and Alternative Medicine*.

[10] Wang MY, Su C (2001). Cancer preventive effect of Morinda citrifolia (Noni). *Annals of the New York Academy of Sciences*.

[11] Singh SK, Rai PK, Jaiswal D, Watal G (2008). Evidence-based critical evaluation of glycemic potential of Cynodon dactylon. *Evidence-Based Complementary and Alternative Medicine*.

[12] Samane S, Noël J, Charrouf Z, Amarouch H, Haddad PS (2006). Insulin-sensitizing and anti-proliferative effects of *Argania spinosa* seed extracts. *Evidence-Based Complementary and Alternative Medicine*.

[13] Wang M-Y, Anderson G, Nowicki D, Jensen J (2008). Hepatic protection by noni fruit juice against CCl4-induced chronic liver damage in female SD rats. *Plant Foods for Human Nutrition*.

[14] Mahesh A, Shaheetha J, Thangadurai D, Rao DM (2009). Protective effect of Indian honey on acetaminophen induced oxidative stress and liver toxicity in rat. *Biologia*.

[15] Scortichini M, Rossi MP (1991). Preliminary in vitro evaluation of the antimicrobial activity of terpenes and terpenoids towards *Erwinia amylovora* (Burrill) Winslow et al. *Journal of Applied Bacteriology*.

[16] Tsuchiya H, Sato M, Miyazaki T (1996). Comparative study on the antibacterial activity of phytochemical flavanones against methicillin-resistant *Staphylococcus aureus*. *Journal of Ethnopharmacology*.

[17] Matsuda H, Li Y, Yamahara J, Yoshikawa M (1999). Inhibition of gastric emptying by triterpene saponin, momordin Ic, in mice: roles of blood glucose, capsaicinsensitive sensory nerves, and central nervous system. *Journal of Pharmacology and Experimental Therapeutics*.

[18] Yoshikawa M, Murakami T, Matsuda H (1997). Medicinal foodstuffs. X. Structures of new triterpene glycosides, gymnemosides-c, -d, -e, and -f, from the leaves of Gymnema sylvestre R. BR.: influence of gymnema glycosides on glucose uptake in rat small intestinal fragments. *Chemical and Pharmaceutical Bulletin*.

[19] Norberg Å, Nguyen KH, Liepinsh E (2004). A novel insulin-releasing substance, phanoside, from the plant Gynostemma pentaphyllum. *Journal of Biological Chemistry*.

[20] Chen J, Li WL, Wu JL, Ren BR, Zhang HQ (2008). Hypoglycemic effects of a sesquiterpene glycoside isolated from leaves of loquat (Eriobotrya japonica (Thunb.) Lindl.). *Phytomedicine*.

[21] De Tommasi N, De Simone F, Cirino G, Cicala C, Pizza C (1991). Hypoglycemic effects of sesquiterpene glycosides and polyhydroxylated triterpenoids of Eriobotrya japonica. *Planta Medica*.

